# Dual targeting of HSP90 and BCL-2 in breast cancer cells using inhibitors BIIB021 and ABT-263

**DOI:** 10.1007/s10549-024-07587-1

**Published:** 2025-01-09

**Authors:** Nazan Gökşen Tosun, Özlem Kaplan

**Affiliations:** 1 Tokat Vocational School of Health Services, Department of Medical Services and Techniques, Tokat Gaziosmanpaşa University, Tokat, Turkey; 2 Rafet Kayış Faculty of Engineering, Department of Genetics and Bioengineering, Alanya Alaaddin Keykubat University, Antalya, Turkey

**Keywords:** Hsp90 inhibition, Navitoclax, BIIB021, Combination treatment, Breast cancer

## Abstract

**Purpose:**

The incidence of breast cancer has been increasing in recent years, and monotherapy approaches are not sufficient alone in the treatment of breast cancer. In the combined therapy approach, combining two or three different agents in lower doses can mitigate the side effects on living cells and tissues caused by high doses of chemical agents used alone. ABT-263 (navitoclax), a clinically tested Bcl-2 family protein inhibitor, has shown limited success in clinical trials due to the development of resistance to monotherapy in breast cancer cells. This resistance shows that monotherapy approaches are inadequate and more effective treatment strategies are needed. It is the ability of HSP90 inhibitors to destabilize many oncoproteins that are critical for the survival of cancer cells. This study aimed to examine the anticancer activity of the combination of ABT-263 with BIIB021, a new generation HSP90 inhibitor, on two widely used breast cancer cell lines: MCF-7 (ER-positive) and MDA-MB-231 (triple-negative breast cancer, TNBC). These cell lines were selected to represent distinct breast cancer subtypes with different molecular characteristics and clinical behaviors.

**Methods:**

Single and combined cytotoxic effects of this agents on MCF-7 and MDA-MB-231 breast cancer cell lines were determined using the MTT cell viability test. The combined use of these two agents showed a synergistic effect, and this effect was assigned using the Chou and Talalay method. mRNA and protein levels of apoptosis-related genes Bax, Bcl-2, Casp9, and Heat Shock Proteins HSP27, HSP70, and HSP90 were analyzed using Quantitative Real-Time Polymerase Chain Reaction (qRT-PCR) and Western Blotting, respectively.

**Results:**

The cytotoxicity analysis, combined with the application of the Chou-Talalay method, demonstrated that the BIIB021 and ABT-263 combination exhibited significantly greater anticancer activity compared to the individual effects of either BIIB021 or ABT-263 in breast cancer cell lines. The analysis of mRNA and protein levels indicated that the BIIB021+ABT-263 combination may have triggered the intrinsic apoptotic pathway in breast cancer cells.

**Conclusion:**

This study showed that co-administration of ABT-263 and BIIB021 agents exhibited synergistic cytotoxic effects and increased the expression of apoptosis-related genes in breast cancer cell lines

**Graphical abstract:**

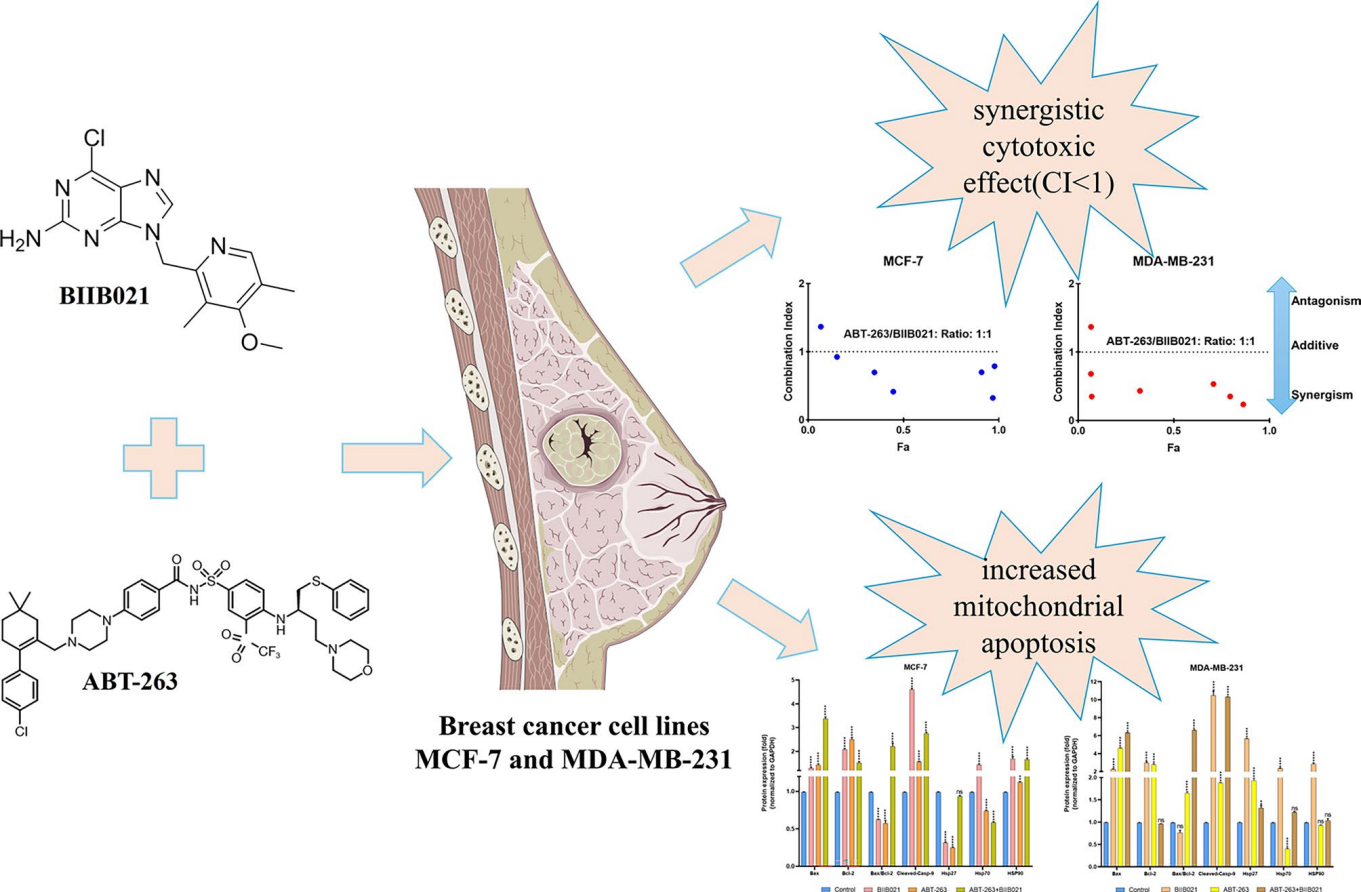

## Introduction

Breast cancer is characterized by the uncontrolled proliferation of epithelial cells within the breast tissue and is the most frequently diagnosed cancer type among women globally [1]. In 2020, an estimated 2.3 million new cases of breast cancer were reported, which contributed to approximately 16% of cancer-related deaths in women, with around 685.000 deaths recorded [2]. Breast cancer has a complex origin, involving various signaling pathways whose biological mechanisms have not yet been fully elucidated [3, 4]. Conventional treatments include surgery as the primary approach, often followed by chemotherapy or radiotherapy. In cases where tumor size is substantial, preoperative systemic therapies such as chemotherapy are employed to reduce the tumor volume, enabling breast-conserving surgery and minimizing the need for extensive lymph node dissection [5]. Since these treatment methods are not target-specific, the side effects they cause in healthy cells and tissues cause mental and physiological problems in patients [6].

Navitoclax (as known ABT-263) is an inhibitor of the B-cell lymphoma 2 (BCL-2) protein family, a regulator of programmed cell death. The BCL-2 family consists of anti-apoptotic and pro-apoptotic proteins that determine the survival of cells. BCL-2 family inhibitors are pharmaceuticals studied in the treatment of cancer, fibrotic, and autoimmune diseases with the approach of strengthening the intrinsic apoptotic pathway by targeting anti-apoptotic proteins [7, 8]. ABT-263 is an oral drug and has been tested alone and in combination with drugs targeting different mechanisms in various types of cancer [9]. It has been a subject of numerous in vitro, preclinical, and phase I and phase II clinical studies due to its potential effects.[8, 10–12]. Despite its promising potential, ABT-263 demonstrated limited efficacy in clinical trials, attributed to the development of resistance to monotherapy specifically in breast cancer cells [13]. Combination therapy is one of the most commonly used approaches in cancer treatment and aims to increase treatment effectiveness by using different drugs together [14]. Compared to monotherapy, in this approach, the combined use of pharmaceutical medications targeting other molecular mechanisms increases anticancer effectiveness while overcoming the adverse side effects caused by high doses on healthy cells and tissues [15].

Heat shock protein 90 (Hsp90) is an important molecular chaperone protein responsible for the folding, activity, and stability of client proteins [16] These Hsp90 proteins are known to be overexpressed in the proliferation, survival, and even tumorigenesis of cancer cells [17, 18]. Over 400 HSP90 client proteins have been identified and are involved in a wide variety of important biological activities, including DNA damage repair, hormone receptor activation, signaling cascades, and protein transport [16]. These client proteins include signaling protein (SRC and AKT), transcription factors (TP53 and HIF-1), receptor tyrosine kinases (EGFR, HER2, IGF-1R, and MET), and cell cycle regulatory proteins (CDK4 and CDK6) [19].

In breast cancer cells, some oncogenes often show irregularity in cancer cells, such as Bcl-2, EGFR, and HER-2. Hsp90 plays important roles in the activation and stabilization of the proteins expressed by these oncogenes, making cancer cells resistant to treatment and ensuring their survival [20]. BCL-2 inhibitors, such as ABT-263, increase apoptosis by neutralizing anti-apoptotic proteins, but their efficacy is often limited as monotherapy due to compensatory survival pathways regulated by HSP90 [21]. Simultaneous inhibition of HSP90 and BCL-2 offers a strategy to target these interconnected mechanisms, destabilize oncogenic proteins, and promote apoptosis in resistant breast cancer cells. This dual inhibition approach in breast cancer may potentially improve treatment outcomes by addressing underlying resistance mechanisms. The new generation of Hsp90 inhibitors have been synthesized to prevent the growth and survival of cancer cells by inhibiting Hsp90 protein activity. These new generation agents targeting Hsp90 protein activity have become a promising new approach in cancer treatment [22]. As a result of Hsp90 inhibition, the stability and activation of oncogenic proteins can be impaired, or irregular folding may occur, which can lead to the death of cancer cells [23]. Therefore, in recent years, the treatment effectiveness of Hsp90 inhibitors on various types of cancer has been investigated through in vitro and clinical studies [24, 25]. Hsp90 regulates not only proteins related to tumorigenesis but also the activities of many different proteins. There are studies to develop more target-specific Hsp90 inhibitors by understanding the effects, molecular mechanisms, and off-target effects of Hsp90 inhibitors on cancer cells. Considering all this information, the importance of Hsp90 inhibitors in cancer treatment is an unquestionable fact [23–28].

BIIB021 (3’,5’-dimethyl-4’-methoxy-2’-pyridyl derivative) is the first orally bioavailable drug among the new generation Hsp90 inhibitor. BIIB021 has been investigated to elucidate anti-cancer effectiveness with in vitro, in vivo, and clinical studies [29–31]. Blocking HSP90 ATPase activity using BIIB021 prevents oncogenic client proteins from folding properly. For this reason, BIIB021 is included in the literature as a promising drug with new-generation approaches to cancer treatment [29, 30, 32].

In this study, the effect of the combination of BIIB021 and ABT-263 on breast cancer cell lines MCF-7 and MDA-MB-231 was investigated. Although each of these agents is well established as monotherapy, previous studies have not evaluated the combination of these agents in breast cancer cells. The aim is to use lower doses of both drugs, reduce side effects and at the same time increase anticancer effectiveness. The cytotoxic effect of BIIB021 and ABT-263 on breast cancer cells was analyzed by MTT test, and the possible effect of the combined use of the drugs was revealed by the Chou-Talalay method. Finally, when the drugs were administered to cells alone and in combination, the changes in the expression levels of apoptosis-related genes and heat shock protein genes were investigated by qRT-PCR and Western Blotting.

## Materials and methods

### Materials

Navitoclax (ABT-263) was obtained by MedChemExpress. BIIB021 was purchased from AdooQ® The 3-(4,5-dimethylthiazol-2-yl)−2,5-diphenyltetrazolium bromide (MTT) and Polyvinylidene difluoride (PVDF) membrane (741,260) were provided by Macherey–Nagel. The BCA protein assay kit and RIPA lysis buffer were obtained from Serva. Abcam supplied the following antibodies: Anti-Bax (ab53154), Anti-Bcl-2 (ab59348), anti-Casp-9 (ab25758), Anti-HSP27 (ab5579), anti-HSP70 (ab79852), anti-HSP90 (ab2928), and goat anti-rabbit IgG H&L (HRP) (ab205718). SYBR Green master mix was procured from Bioline. Primers were synthesized by Metabiomics. Favorgen Biotech Corp. provided the total RNA isolation kit. Bio-Rad supplied the cDNA synthesis kits and the enhanced chemiluminescence (ECL) substrate kit (1,705,060). Dulbecco's Modified Eagle's medium (DMEM) High Glucose, penicillin–streptomycin solution, phosphate buffer saline (PBS), fetal bovine serum (FBS), trypsin–EDTA and L-glutamine were obtained from Biological Industries. The MCF-7 and MDA-MB-231 cell lines were purchased from ATCC (American Type Culture Collection).

### Cell culture

Breast cancer cell lines MCF-7 and MDA-MB-231 were cultured in DMEM High Glucose medium supplemented with 10% FBS and 0.1% penicillin–streptomycin. The MCF-7 cell line is an estrogen receptor-positive (ER+) human breast cancer cell line. MDA-MB-231 is a triple-negative breast cancer (TNBC) cell line, which lacks estrogen, progesterone, and HER2 receptors [4]. The cell lines were then incubated at 37 °C in a humidified atmosphere with 5% CO_2_. Upon reaching 90% confluence, the cells were passaged and these cell lines were passaged no more than 10 times [33].

### Cell viability assay

The in vitro cytotoxic effects of BIIB021 and ABT-263 on breast cancer cell lines were determined by MTT test. The breast cancer cell lines were seeded in 96-well culture plates (5 × 10^4^ cells per well) and treated with BIIB021 and ABT-263 for 24 and 48 h at concentrations ranging from 1.56 to 100 nM and 1.56 to 100 µM, respectively. After the incubation period, the culture medium was removed, and then each well was supplemented with MTT solution (5 mg/mL), followed by an additional incubation at 37 °C for 3 h. Subsequently, the formazan products resulting from the reduction of MTT by viable cells were dissolved in DMSO, and their absorbance was measured at a wavelength of 570 nm. The determination of IC_50_ values were performed using GraphPad Prism 8.01 software. At least three independent experiments were performed [34]. CompuSyn software, based on the median-effect equation, is widely utilized for evaluating drug combination effectiveness through the calculation of combination indices (CIs). This program analyzes dose-dependent drug interactions in detail, categorizing effects as follows: CI = 1 indicates an additive effect, CI < 1 denotes a synergistic effect, and CI > 1 suggests an antagonistic effect [35]. The drug interactions between ABT-263 and BIIB021 were assessed in CompuSyn, using certain concentrations.

### Quantitative RT-PCR

MCF-7 and MDA-MB-231 cells were treated with BIIB021, ABT-263 and BIIB021 + ABT-263 for 48 h at the calculated IC_50_ values. RT-PCR was used to investigate the change in the expression of the apoptosis-related proteins Bax, Bcl-2, Casp-9 genes, and heat shock proteins Hsp27, Hsp70, Hsp90 genes in these cells. RT-PCR was performed according to the protocol detailed previously [4]. Briefly, total RNA was isolated from cells treated with both individual drugs and their combinations, and cDNA synthesis was subsequently carried out using the isolated RNA samples. A qRT-PCR assay using the Bio-Rad CFX96TM system was performed with a 25 µL reaction volume. The reaction mixture included 1 µL cDNA, 1 µL primer mix (10 mM stock), 11.5 µL SYBR Green qPCR master mix, and 10.5 µL RNase-free water. The thermal cycling conditions consisted of an initial denaturation at 95 °C for 10 min, followed by 40 cycles of denaturation at 95 °C for 15 s and annealing/extension at 60 °C for 60 s. The primer sequences are shown in Table 1. Gene expression levels were analyzed using the 2^−ΔΔCt^ method. The data were normalized using GAPDH as the housekeeping gene. At least three independent experiments were performed.

**Table 1:** The primer sequences of genes (F: Forward, R: Reverse)

Primer		Sequence
*Bax*	F	5’-TCAGGATGCGTCCACCAAGAAG-3’
	R	5’-TGTGTCCACGGCGGCAATCATC-3’
*Bcl-2*	F	5’-ATCGCCCTGTGGATGACTGAGT-3’
	R	5’-GCCAGGAGAAATCAAACAGAGGC-3’
*Casp-9*	F	5’-GTTTGAGGACCTTCGACCAGCT-3’
	R	5’-CAACGTACCAGGAGCCACTCTT-3’
*Hsp27*	F	5’-CTGACGGTCAAGACCAAGGATG-3’
	R	5’-GTGTATTTCCGCGTGAAGCACC-3’
*Hsp70*	F	5’-ACCTTCGACGTGTCCATCCTGA-3’
	R	5’-TCCTCCACGAAGTGGTTCACCA-3’
*Hsp90*	F	5’-TCTGCCTCTGGTGATGAGATGG-3’
	R	5’-CGTTCCACAAAGGCTGAGTTAGC-3’
*Gapdh*	F	5’-GTCTCCTCTGACTTCAACAGCG-3’
	R	5’-ACCACCCTGTTGCTGTAGCCAA-3’

### Western blotting

The Western blotting method was employed to assess changes in the expression levels of apoptosis-related proteins (Bax, Bcl-2, and Casp-9) and heat shock proteins (Hsp27, Hsp70, and Hsp90) in MCF-7 and MDA-MB-231 cells treated with BIIB021, ABT263, and BIIB021 + ABT263 at their respective IC_50_ doses for 48 h. Following the previously described protocol [15], the cells were harvested by trypsin treatment, and total protein isolation was performed with RIPA buffer. The concentration of total protein in the lysates was determined using the BCA protein assay kit. Subsequently, 30 μg of proteins per well were separated by 12% SDS-PAGE gel electrophoresis and transferred onto PVDF membranes using a Trans-blot turbo transfer device from Bio-Rad. The membranes were blocked using 5% non-fat milk in Tris-buffered saline/Tween 20 (TBST) for 1 h. The primary antibodies, including anti-HSP27 (1:1000), anti-HSP70 (1:500), anti-HSP90 (1:500), anti-Bax (1:1000), anti-Bcl-2 (1:500), and anti-Casp-9 (1:500), were then incubated overnight at 4 °C. After washing with TBST, membranes were incubated with a secondary antibody [goat anti-rabbit IgG H&L (HRP)] (1:10,000) for 1 h at room temperature The data were normalized relative to GAPDH (anti-GAPDH primary antibody diluted 1:5000). Protein bands were visualized using ChemiDoc™ imaging equipment (Bio-Rad) and an ECL substrate. ImageLab 6.1 software was used to analyze protein expression levels. At least two independent experiments were performed.

### Protein–protein interaction network analysis and pathway enrichment analysis

In silico analyzes were performed to understand in more detail the molecular pathway of the effect of co-administration of two drugs. First, a protein–protein interaction network consisting of genes associated with apoptosis and six genes selected from the heat shock protein family was created. This was accomplished using the Search Tool for the Retrieval of Interacting Genes (STRING) with the Cytoscape v3.10.2 software for assessing gene interactions. Pathway enrichment analysis Kyoto Encyclopedia of Genes and Genomes (KEGG) ve Gene Ontology (GO) biological process analyzes were performed using the ClueGo v2.5.10 and CluePedia v1.5.10 platform via Cytoscape v3.10.2 software.

### Statistical analysis

GraphPad Prism 8.0 software was used to perform a two-way ANOVA test with Sidak and Dunnett tests. Statistical significance was defined as probability values of p < 0.05 To evaluate the combined effect and calculate the combination index of the drug combination, the Chou-Talalay method was performed using CompuSyn software version 1.0.

## Results and discussion

**Fig. 1:**
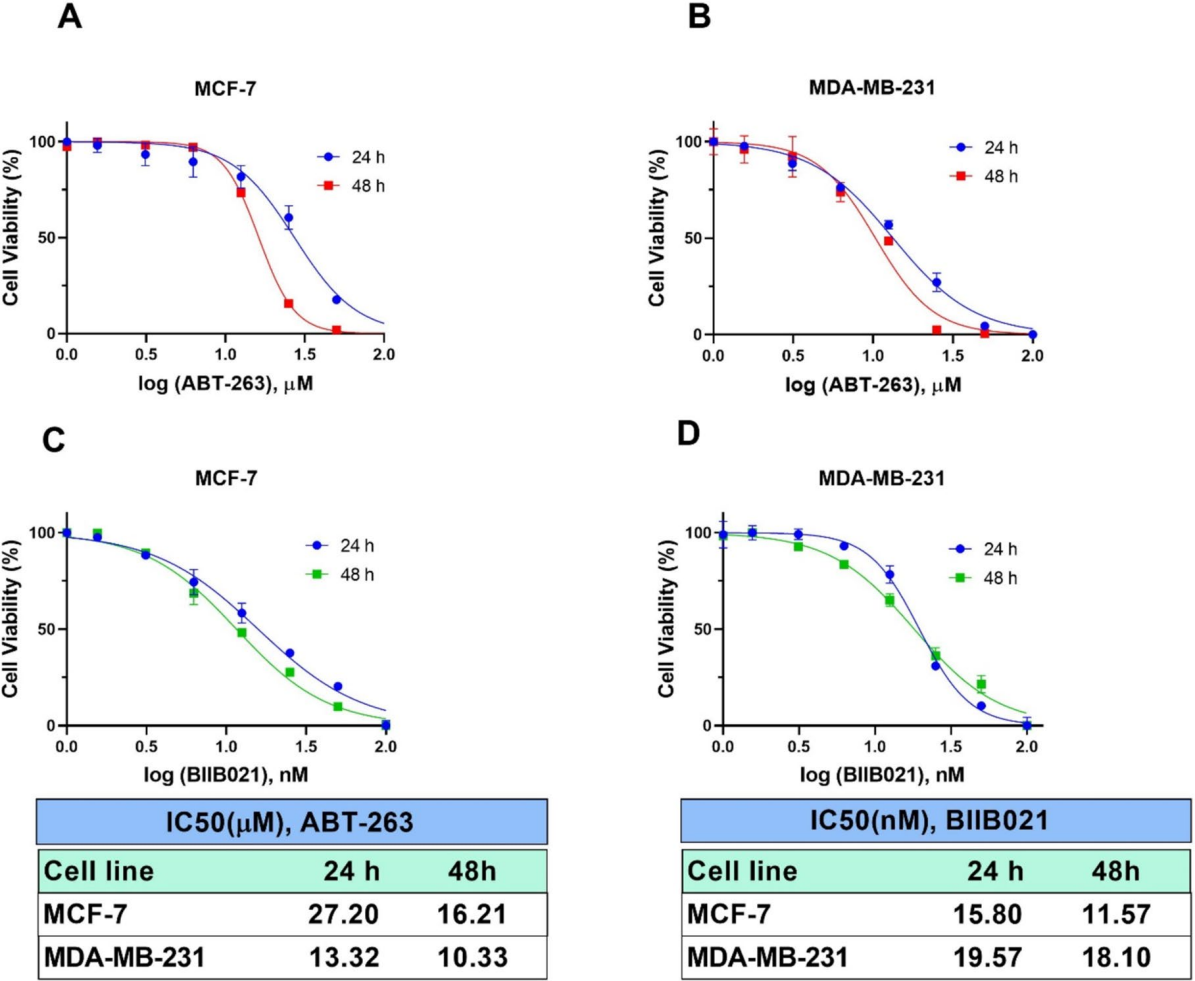
The dose- and time-dependent effects of ABT-263 and BIIB021 on the viability of MCF-7 and MDA-MB-231 cell lines, along with the calculated IC_50_ values of ABT-263 and BIIB021. The cell viability of MCF-7 (**A**) and MDA-MB-231 (**B**) cells after exposure to various doses of ABT-263 after 24 and 48 h. The cell viability of MCF-7 (**C**) and MDA-MB-231 (**D**) cells after exposure to various doses of BIIB021 after 24 and 48 h

In this study, the effects of HSP90 inhibitor BIIB021 and BCL-2 inhibitor ABT-263 on breast cancer cell lines MCF-7 and MDA-MB-231 were investigated. The cytotoxic effects of BIIB021, ABT-263 and BIIB021 + ABT-263 combination on MCF-7 and MDA-MB-231 cell lines were evaluated by MTT test. BIIB021 and ABT-263 exhibited dose- and time- dependent cytotoxic effects on both cell lines. The IC_50_ values for BIIB021 and ABT-263 were 11.57 nM and 16.21 µM, respectively, in MCF-7 cells, and 10.58 nM and 10.33 µM, respectively, in MDA-MB-231 cells (Fig. 1). MDA-MB-231 cells were more sensitive to both drugs compared to MCF-7 cells. Previous studies have reported that ABT-263 may be a potential treatment option for breast cancer cells, but resistance to monotherapy occurred in clinical trials. Additionally, monotherapy with BCL-2 family inhibitors has not induced cell death in some breast cancer cell lines [7, 36, 37]. Lee et al. revealed that ABT-263 has different potencies on MDA-MB 231 and MCF-7 cell lines. While ABT-263 treatment led to resistance in MCF-7 cells against ABT-263, it induced cell death in MDA-MB-231 cells. The findings of this study were compared with the study by Lee et al. published results, further confirming that a 48-h treatment period was more effective. Consistent with this research, MCF-7 cells demonstrated greater resistance to ABT-263 compared to MDA-MB-231 cells. In the study by Lee et al., MCF-7 and MDA-MB-231 cells were treated with up to 5 µM ABT-263 for 48 h, resulting in approximately 20% cell death in MCF-7 cells and 85% in MDA-MB-231 cells. By contrast, the present study reported IC50 values of 16.21 µM for MCF-7 cells and 10.33 µM for MDA-MB-231 cells, highlighting differences in sensitivity while maintaining the overall trend observed in both studies. However, the combination treatment of ABT-263 and everolimus (a survivin inhibitor) was effective in inducing an intrinsic apoptotic pathway in MCF-7 cells [13].

**Fig. 2:**
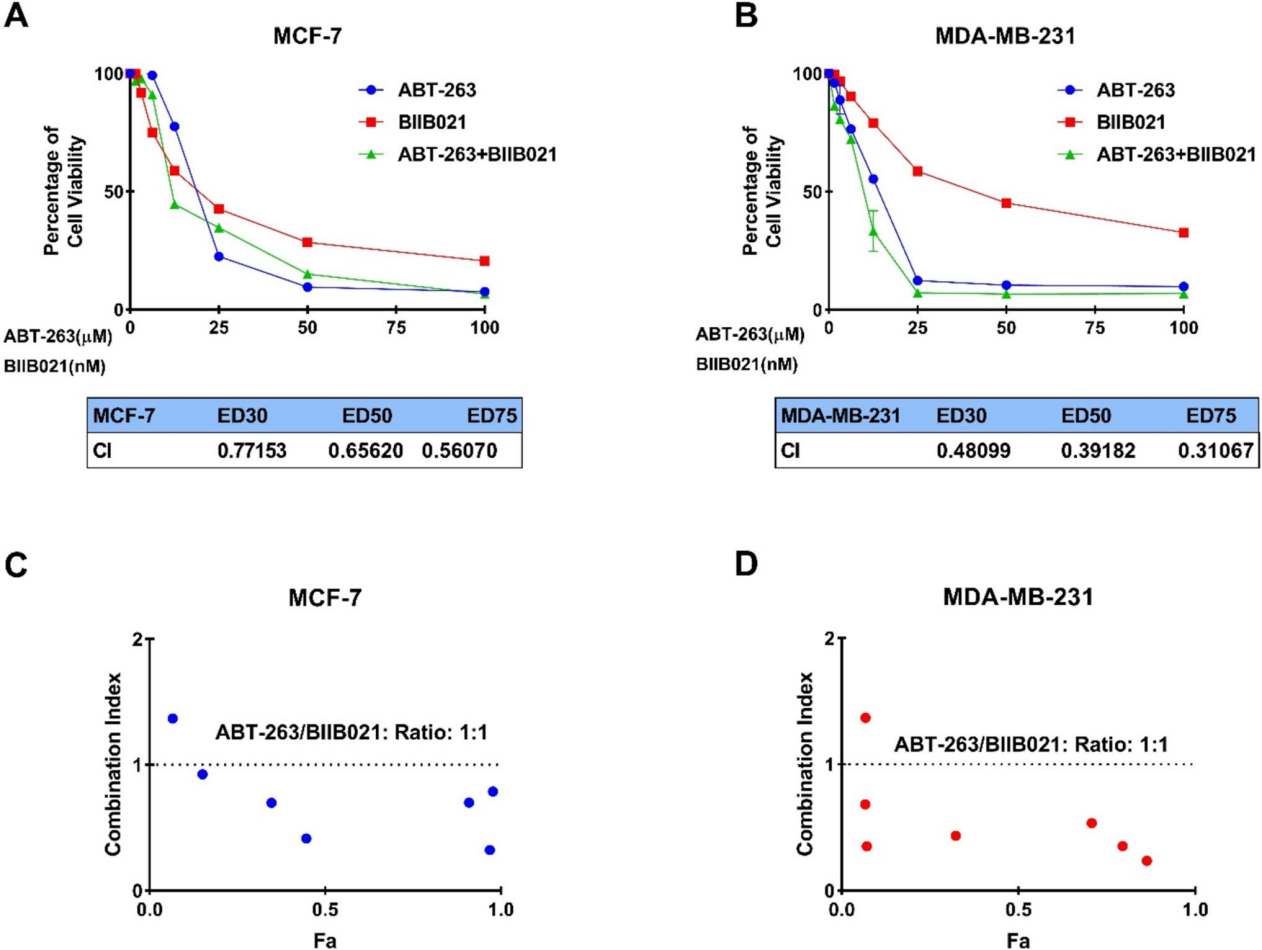
The combined cytotoxic effects of ABT-263 and BIIB021 in MCF-7 (**A**) and MDA-MB-231 (**B**) cancer cell lines. Fraction Affected (Fa) vs Combination Index (CI) plots derived from Chou-Talalay median-effect analysis for MCF-7 (**C**) and MDA-MB-231 (**D**) cell lines, respectively. These plots illustrate the relationship between the fraction of affected cells (Fa) and the combination index (CI), providing insights into the synergistic, additive, or antagonistic effects of the drug combination. Additionally, the terms ED30, ED50, and ED75 denote the doses at which 30, 50, and 75% of cells or organisms are effectively inhibited

The dose-dependent cell viability was assessed at two-time points: 24 and 48 h. Comparative analysis of the IC_50_ values revealed that 48 h of incubation resulted in greater cytotoxic efficacy in both MCF-7 and MDA-MB-231 cell lines. Therefore, the combined effect of the ABT-263 and BIIB021 was assessed within 48 h on MCF-7 and MDA-MB-231 cells using the Chou-Talalay method. The Chou and Talalay method emerge as a fundamental approach in the examination of drug combinations. Its framework is based on the median impact equation derived from the law of mass action, which combines both single- and multi-asset scenarios. The adoption of the median effect equation, based on mass action principles, has accelerated its practical application in research [38, 39]. The Chou and Talalay approach gained popularity with the release of the CompuSyn software in 2005 [40]. CompuSyn's integration with the Chou and Talalay method significantly improved the analysis process, enabling comprehensive evaluations covering dose ranges, combination ratios, design layouts, and computerized simulations of drug interactions. This program represents a powerful tool for uncovering the complexity of drug combinations [41]. The research proposes that the combined effects of two agents, whether synergistic, additive, or antagonistic, can be characterized as follows: A combination index (CI) value is equal to 1, it indicates an additive effect; if it is greater than 1, it suggests an antagonistic effect, and if it is less than 1, it implies a synergistic effect [35]. In this study, the CI values were calculated as 0.65620 and 0.39182 for MCF-7 and MDA-MB-231 cells, respectively. Therefore, co-administration of ABT-263 and BIIB021 showed synergistic cytotoxic effect in both MCF-7 and MDA-MB-231 cell lines. The CompuSyn software calculated total dose values corresponding to synergistic effects for both cell lines, and the results are summarized in Fig. 2. Specifically, the combination indices determined as CI = 0.65620 for MCF-7 cells and CI = 0.39182 for MDA-MB-231 cells, with corresponding total dose values of 18.0812 and 7.55305 µM, respectively. The concentration-dependent cell viability graphs shown in Fig. 2A, B were compared to these total dose values. The results revealed that the total doses identified by CompuSyn, where synergy occurs, were more effective than the doses of the drugs when used individually. This highlights the potential enhanced efficacy of the combined treatment for both cell lines.

Previous studies have shown that co-administration of HSP90 inhibitors with various anticancer agents, including Bcl-2 inhibitors, exhibits synergistic effects. An HSP90 inhibitor, Ganetespib, and the BCL-2 inhibitor, ABT-199, were found to synergistically inhibit cell proliferation, with 8 nM Ganetespib combined with 4 µM ABT-199 in the cervical cancer cell line HeLa cells [42]. In our previous study, the traditional chemotherapeutic agent doxorubicin and the new generation HSP90 inhibitor MPC-3100 showed a synergistic effect on breast cancer cell lines MCF-7 and MDA-MB-231 and induced apoptosis [4]. Subaiea et al. reported that the co-administration of Ganetespib with the chemotherapeutic agent Methotrexate demonstrated effective inhibition of lung cancer cell proliferation [43].

**Fig. 3:**
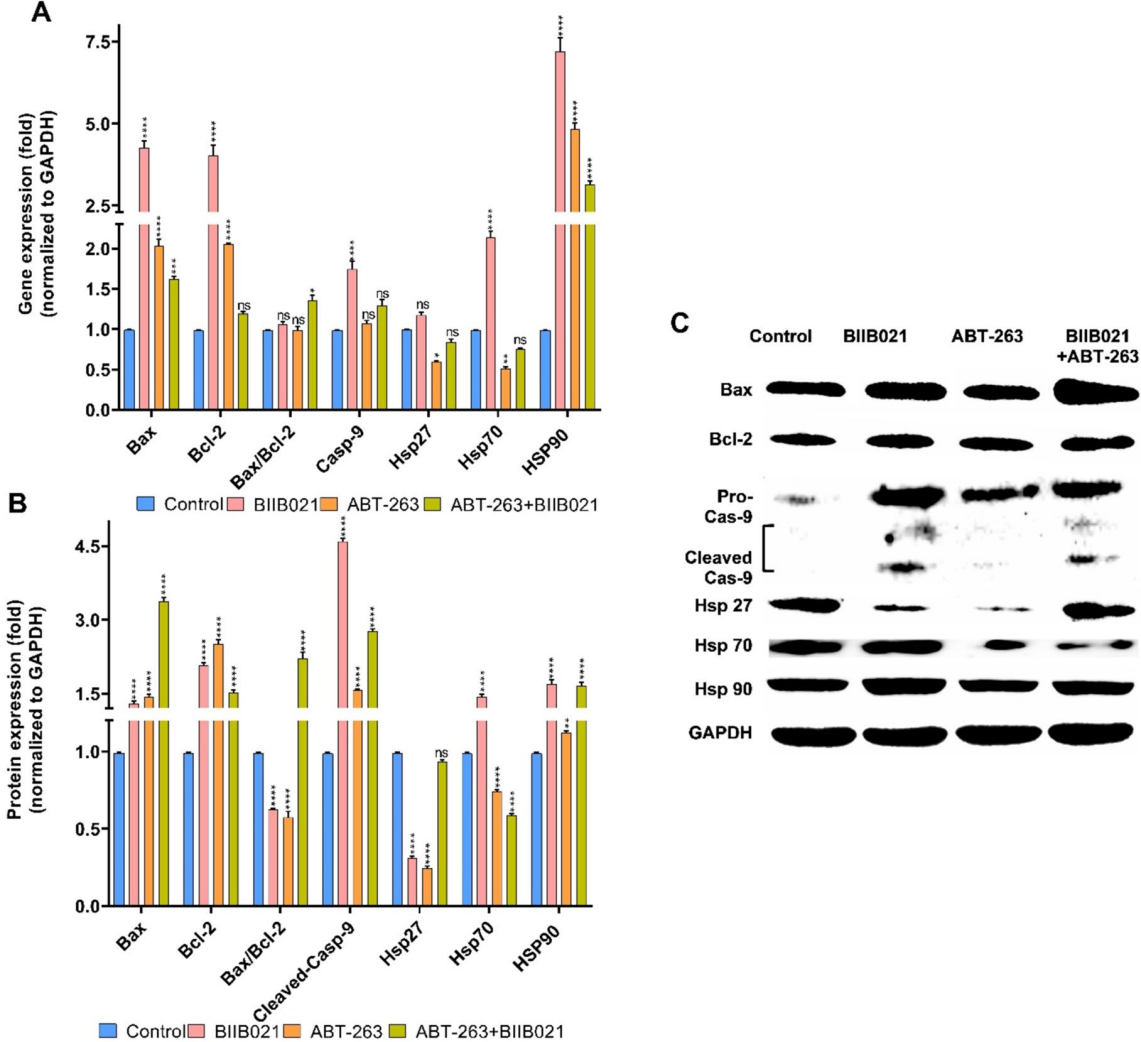
Changes in the expression of apoptosis-related proteins (Bax, Bcl-2, Casp-9) and heat shock proteins (Hsp27, HSP70, HSP90) in MCF-7 cells after a 48-h treatment with BIIB021, ABT-263, and BIIB021 + ABT-263. Gene expression (**A**) and protein expression (**B**) changes in apoptosis-related proteins (Bax, Bcl-2, pro-Casp-9) and HSPs (HSP27, HSP70, HSP90), the abundance of apoptosis-related proteins and HSPs in MCF-7 (**C**). (*p < 0.05, **p < 0.01, ***p < 0.001, and ****p < 0.0001)

To unravel the mechanism underlying the cytotoxic effects of ABT-263, BIIB021, and their combination on breast cancer cell lines, the mRNA and protein expression levels of apoptosis-related proteins (Bax, Bcl-2, Casp-9) and heat shock proteins (Hsp27, HSP70, HSP90) were analyzed using RT-PCR and Western Blotting. Figure 3 illustrates the changes in mRNA and protein levels of apoptosis-related proteins and HSPs in MCF-7 cells treated with BIIB021, ABT-263 and BIIB021 + ABT-263. According to Fig. 3A, BIIB021 resulted in increased mRNA levels of Bax, Bcl-2, and Casp-9 when administered to MCF-7 cells. The increasing trend in the Bax/Bcl-2 ratio, which is an indicator of the susceptibility of cells to apoptosis, was not statistically significant. The HSP90 inhibitor BIIB021 induced a significant increase in HSP70 and HSP90 mRNA levels in MCF-7 cells, while the slight increase in HSP27 was statistically insignificant. When the changes in the protein levels of these genes are examined (Fig. 3B, C), it was observed that the protein amounts of Bax and Bcl-2 increased in parallel with their mRNA levels. However, the Bax/Bcl-2 ratio decreased at the protein level when compared to control cells, despite the increase observed at the mRNA level. There was a significant increase in the level of cleaved Casp-9. While changes in HSP70 and HSP90 protein levels increased in parallel with mRNA levels, HSP27 protein level decreased. In MCF-7 cells exposed to the BCL-2 family inhibitor ABT-263, there was an increase in the mRNA and protein levels of Bax and Bcl-2. Although the increase in Casp-9 mRNA levels was statistically insignificant, a slight increment was observed, and this increase was also present in cleaved Casp-9 protein levels. These findings suggest that ABT-263 could induce apoptosis in MCF-7 cells. Examining the HSP mRNA and protein changes in MCF-7 cells subjected to ABT-263 treatment revealed a decrease in HSP27 and HSP70 levels, while an increase was observed in HSP90 levels. The co-administration of ABT263 + BIIB021 in MCF-7 cells resulted in an increase in the Bax/Bcl-2 ratio and cleaved-Casp-9 at both mRNA and protein levels, suggesting that apoptosis-related pathways may have been influenced. Analyzing HSPs, there was a decrease in both HSP27 and HSP70, while HSP90 exhibited an increase in both mRNA and protein expression levels.

**Fig. 4:**
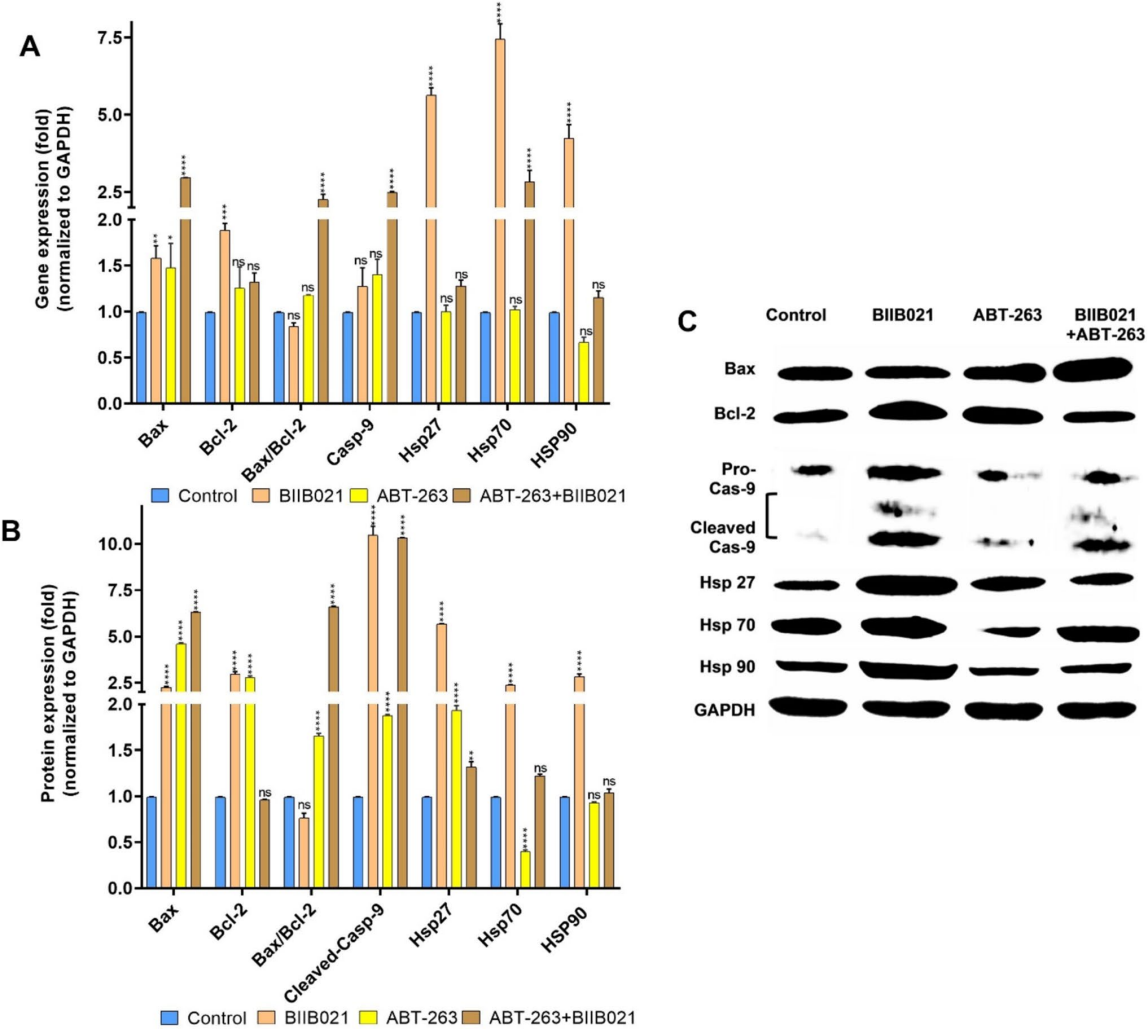
Changes in the expression of apoptosis-related proteins (Bax, Bcl-2, Casp-9) and heat shock proteins (Hsp27, HSP70, HSP90) in MDA-MB-231 cells after a 48 h treatment with BIIB021, ABT-263, and BIIB021 + ABT-263. Gene expression (**A**) and protein expression (**B**) apoptosis-related proteins (Bax, Bcl-2, pro-Casp-9) and HSPs (HSP27, HSP70, HSP90), the abundance of apoptosis-related proteins and HSPs in MCF-7 (**C**). (*p < 0.05, **p < 0.01, ***p < 0.001, and ****p < 0.0001)

Figure 4 depicts the changes in mRNA and protein levels of apoptosis-related proteins and HSPs in MDA-MB-231 cells treated with BIIB021, ABT-263 and BIIB021 + ABT-263. MDA-MB-231 cells treated with BIIB021 exhibited a similar profile to MCF-7 cells. Bax, Bcl-2, Cleaved-Casp-9, HSP70, and HSP90 increased. In contrast to MCF-7 cells, an increase in HSP27 protein was observed in MDA-MB-231 cells. The data indicate that each cell line responds differently to BIIB021 treatment. The upregulation of HSP70 in both breast cancer cell lines upon inhibition of HSP90 activity by BIIB021 can be linked to the separation of monomers of heat shock factor 1 (HSF1) from HSP90. This separation triggers the trimerization of HSF1, its translocation to the nucleus, and the subsequent transcriptional activation of HSP70 [44, 45]. MDA-MB-231 cells treated with ABT-263 exhibited a distinct protein expression profile compared to MCF-7. In these cells, the Bax/Bcl-2 ratio and cleaved Casp-9 increased, suggesting a potential involvement of apoptosis-related pathways. HSP27 increased, HSP70 decreased, and HSP90 remained unchanged. The differential protein expression profile in cells exposed to ABT-263 may contribute to the heightened sensitivity of MDA-MB-231 cells compared to the lower sensitivity observed in MCF-7. When BIIB021 and ABT-263 were co-administered, apoptotic markers, including the Bax/Bcl-2 ratio and cleaved-Casp-9, increased in MDA-MB-231 cells. The protein level of HSP27, HSP70, and HSP90 increased, but the slight increase in HSP70 and HSP90 protein levels was not statistically significant.

**Fig. 5:**
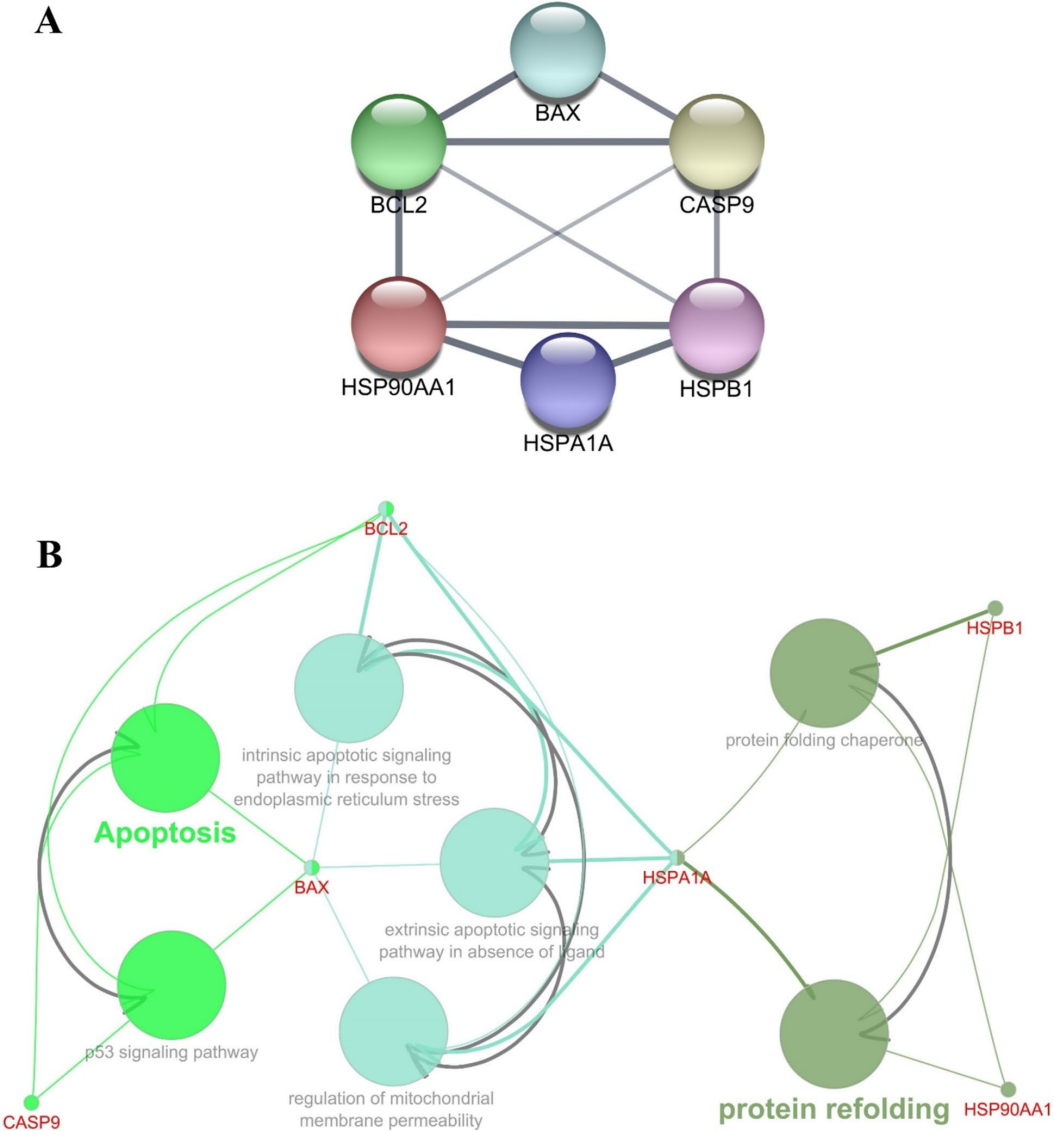
**A** Protein–protein interaction network analysis of heat shock proteins (HSP90, HSP70, HSP27) and apoptosis-related proteins (Bax, Bcl-2, Casp-9) using the STRING database and visualized with Cytoscape v3.10.2. This network highlights the interactions and potential functional relationships between these proteins. **B** Pathway enrichment analysis using KEGG and Gene Ontology (GO) biological processes performed with ClueGo v2.5.10 and CluePedia v1.5.10 via Cytoscape v3.10.2

To summarize the effects of the study groups on mRNA and protein levels, it was observed that BIIB021 increased Bax, Bcl-2 and Casp-9 mRNA levels in MCF-7 and MDA-MB-231 cells. However, an increase in HSP27 protein was observed in MDA-MB-231 cells, unlike MCF-7 cells. These findings indicate that both cell lines respond differently to BIIB021. HSP70 was determined to be upregulated in both breast cancer cell lines by inhibiting HSP90 activity by BIIB021. With the use of ABT-263, an increase in Bax and Bcl-2 mRNA and protein levels were observed in MCF-7 and MDA-MB-231 cells. However, it was determined that the increase in the Bax/Bcl-2 ratio and cleaved Casp-9 levels, which are associated with apoptosis-related pathways, was more pronounced in MDA-MB-231 cells. Finally, the combination of BIIB021 and ABT-263 appeared to enhance apoptosis-related gene expression in MCF-7 and MDA-MB-231 cells. This combination led to an increase in the Bax/Bcl-2 ratio at both mRNA and protein levels in both cell lines and elevated cleaved Casp-9 levels. These findings suggest that the drug combination may influence apoptosis-related pathways, potentially activating caspases and contributing to programmed cell death processes for maintaining tissue homeostasis, eliminating damaged cells, and regulating development. A complex protein network including pro-apoptotic proteins (e.g., Bax and Casp-9) and anti-apoptotic factors (e.g., Bcl-2) closely regulates apoptosis [46]. In addition, in order to have a deeper understanding of the functional relationship between the two drugs and the roles and pathways of the analyzed genes, the interactions of these proteins and molecular pathway analyses were performed (Fig. 5A, B). Figure 5A illustrates the interaction network between apoptosis-related proteins (Bax, Bcl-2, Casp-9) and heat shock proteins (HSP90 (HSP90AA1), HSP70 (HSPA1A), HSP27 (HSPB1)). Figure 5B shows the biological processes these proteins regulate. Although HSP90 inhibitors have limited clinical use due to their restricted activity, their ability to target multiple pathways, including various apoptotic routes, makes them potential candidates for combination therapies. HSP90 inhibitors promote the proteasomal degradation of HSP90 client proteins such as IGF1R, AKT, EGFR, IKK, RAF-1, c-KIT, NPM-ALK, v-SRC, and P53, thereby inhibiting AKT and MAPK signaling. Given that NF-kB activation is known to induce anti-apoptotic effects, blocking NF-kB may enhance the synergistic anti-cancer effects when combined with BCL-2 inhibitors [21]. Several studies have reported that HSP90 inhibitors can induce apoptosis by decreasing anti-apoptotic proteins and increasing pro-apoptotic proteins [47]. HSP70 (HSPA1A) has been demonstrated to influence both intrinsic and extrinsic apoptotic pathways. In the TNF-α-induced apoptosis model, the HSP70-CHIP complex suppresses JNK and p38 via increasing the degradation of apoptosis signal-regulated kinase 1. JNK stimulates the intrinsic apoptotic pathway by releasing cytochrome c from mitochondria. JNK-dependent apoptosis requires the activation of Bax and Bid via p53. Furthermore, HSP70 suppresses the apoptosis-inducing factor (AIF), which is essential for DNA degradation. In the extrinsic pathway, HSP70 interacts with TNF-related apoptosis-inducing ligand receptors (TRAIL-R1 and TRAIL-R2) to block the development of the death-inducing signaling complex (DISC). This may also hinder caspase-8 from cleaving Bid. Thus, HSP70 reduces apoptosis by inhibiting JNK and AIF and interacting with death receptors [48]. In this study, the combination of BIIB021 and ABT-263 in both cell lines may have played a role in driving the cells to apoptosis by reducing the HSP70 expression level. HSP70 can play a pivotal role in regulating apoptosis by interfering with the assembly of the apoptosome, a critical structure in the intrinsic apoptotic pathway. Specifically, HSP70 interacts with Apaf-1, inhibiting its ability to recruit and activate procaspase-9, thereby preventing the formation of an active apoptosome complex. This disruption hinders the subsequent cascade of events leading to apoptosis [49]. In the context of this study, the observed reduction in HSP70 levels following the combination treatment of BIIB021 and ABT-263 may have alleviated this inhibition, allowing for the enhanced activation of caspase-9, as evidenced by the increase in cleaved Casp-9 levels. Given the central role of Casp-9 in propagating the intrinsic apoptotic pathway through downstream caspase activation, these findings highlight a potential mechanism by which the combination treatment promotes apoptosis in the cell lines.

HSP27 (HSPB1) supports the activation of Akt (protein kinase B). Akt regulates cell survival signals and inhibits apoptosis. The activation of Akt leads to the inhibition of apoptotic proteins (such as Bax and Bad) and the activation of anti-apoptotic proteins (such as Bcl-2). HSP27 also inhibits JNK activity [50]. Inhibition of HSP90 and Bcl-2 proteins causes other HSP proteins, including HSP90, HSP70, and HSP27, to alter the expression of apoptotic and pro-apoptotic proteins, leading cells to undergo apoptosis.

In this study, the impact of BIIB021, ABT-263, and BIIB021 + ABT-263 combination on the expression of genes involved in the mitochondrial intrinsic apoptotic pathway was examined in breast cancer cell lines. The BIIB021 + ABT-263 combination significantly elevated the Bax/Bcl-2 expression ratio in both MCF-7 and MDA-MB-231 cells compared to controls and individual substances. Bax, through interaction with anti-apoptotic Bcl-2 and Bcl-xL, orchestrates the intrinsic apoptotic pathway. Overexpression of Bax promotes Cyt-c release from the mitochondria to the cytosol and permeabilization of the mitochondrial outer membrane, but overexpression of Bcl-2 may inhibit this process. Following that, Cyt-c binds to Apaf-1 to form the apoptosome complex, which initiates cell death by activating Casp-3 and/or Casp-7. The apoptosome complex facilitates the activation of Casp-9, leading to the occurrence of apoptosis [51]. Hsp90 overexpression inhibits the apoptotic process, allowing cancer cells to survive. As a result, inhibiting Hsp90 chaperone function has become an important technique in cancer research [19]. Specifically, preclinical studies have demonstrated the effectiveness of BIIB021, a promising candidate Hsp90 inhibitor, and it is now being included in clinical trials for breast cancer, Kaposi's sarcoma, and gastrointestinal stromal tumors [32]. BIIB021 inhibits HSP90 ATPase activity by interfering with the correct folding of oncogenic client proteins. Experimental studies reveal that BIIB021 exhibits a higher affinity for the NTD region of HSP90 compared to other inhibitors [30]. Therefore, BIIB021 plays a crucial role in evaluating a promising HSP90 inhibitor and contributing to the discovery of next-generation cancer drugs. Several studies have investigated the combined effects of BCL-2 inhibitors and HSP90 inhibitors on cancer cells. The Bcl-2 family plays an important role in the intrinsic pathway of apoptosis. Overexpression of Bcl-2 occurs in about 41% of triple-negative breast cancers, 50% of HER2-positive tumors, 85% of ER-positive tumors. The expression of these pro-survival Bcl-2 family proteins is linked to resistance against chemotherapeutic drugs [13]. Wang et al. reported that the combination of BCL-2 inhibitor (–)-gossypol and HSP90 inhibitor 17-AAG in hepatocellular carcinoma cells promoted (–)-gossypol-induced apoptosis by suppressing protective autophagy and reduced Mcl-1 accumulation [52]. Yang et al. reported that the combination of the BCL-2 inhibitor ABT-737 and the HSP90 inhibitor NVP-AUY922 exhibited synergistic anticancer effects in small cell lung cancer (SCLC), especially when ABT-737 showed limited clinical efficacy. The combination treatment synergistically triggered apoptosis in SCLC cells expressing BCL-2. This effect was achieved by reducing the interaction of AKT and ERK with MCL-1 and inhibiting NF-kB activation by NVP-AUY922. The findings suggest that the combination of BCL-2 inhibitor and HSP90 inhibitor may be more effective than each inhibitor, particularly in SCLC expressing BCL-2 [21]. In a recent study on cervical cancer cells, the combination of Ganetespib and ABT-199 demonstrated a synergistic effect, reducing cell proliferation. This combination decreased Hsp90 protein levels and significantly inhibited Hsp90 chaperone activity. Furthermore, it decreased anti-apoptotic markers and increased pro-apoptotic markers by inducing apoptosis. The study revealed that the Ganetespib-ABT-199 combination was more effective in inducing toxicity and apoptosis in cervical cancer cells compared to individual drugs [42].

In this study, the effect of co-administration of BIIB021 and ABT-263 was investigated in breast cancer cell lines, MCF-7 and MDA-MB-231. The combination of BIIB021 and ABT-263 reduced the viability of breast cancer cells by modulating the expression levels of anti-apoptotic and pro-apoptotic genes, thereby stimulating apoptotic signaling pathways. The results of this study suggest that the combination of BIIB021 and ABT-263 may exert a synergistic effect against breast cancer cells by affecting the mitochondrial apoptotic pathway. The combination of BIIB021 and ABT-263 may have cytotoxic effects on breast cancer cells by modulating the mitochondrial intrinsic apoptosis pathway. This study is based on in vitro cell culture models and therefore may not fully reflect the complexity of the tumor microenvironment and interactions with neighboring cells. Breast cancer is divided into different molecular subtypes based on the presence of estrogen receptor (ER), progesterone receptor (PR) and human epidermal growth factor receptor (HER2) expression [53]. In this study, the use of only the luminal A type (ER+/PR+/−/HER2-) cell line MCF-7 and the triple-negative breast cancer (ER-, PR-, HER2-) cell line MDA-MB-231 may not fully represent the heterogeneity of breast cancer subtypes. Investigating the effects of BIIB021 and ABT-263 in a broader range of cell lines, including those derived from different subtypes, would increase the generalizability of our results. Additionally, this study focused on a specific set of apoptosis-related and heat shock proteins. Future studies using in vivo models and broader proteomic approaches are necessary to confirm and extend our findings. Furthermore, future clinical studies are important to confirm the therapeutic potential of the combination of BIIB021 and ABT-263 in breast cancer patients. These studies should also consider long-term treatment effects and the development of potential resistance mechanisms not addressed in our 48-h treatment study. By addressing these limitations in future research, it may provide a better understanding of the therapeutic potential of BIIB021 and ABT-263 combination therapy in the treatment of breast cancer.

## Conclusion

In conclusion, this study highlights the effective anticancer activity of the BIIB021 + ABT-263 combination, exceeding the individual efficacy of BIIB021 or ABT-263 alone in breast cancer cell lines. The combination of BIIB021 + ABT-263 may have stimulated the intrinsic apoptotic pathway in breast cancer cells. The synergistic effects observed in the combination of BIIB021 and ABT-263 suggest a promising therapeutic strategy for the treatment of breast cancer. Our findings elucidate the potential synergistic mechanism between an HSP90 inhibitor BIIB021 and the BCL-2 family inhibitor ABT-263, pointing to the possibility of a more effective therapeutic approach for breast cancer treatment.

Monotherapies in cancer treatment carry with them significant challenges associated with chemotherapy, such as dose-related adverse effects and drug resistance, which are major limitations. Combination therapies have emerged as a superior strategy, as they not only enhance therapeutic efficacy but also allow for dose reduction, thereby minimizing toxicity. The observed synergy in this study underscores the importance of carefully designed combination therapies in maximizing therapeutic benefits and overcoming the limitations of conventional cancer treatments. In this study, the promising effect of the BIIB021 + ABT-263 combination therapy was demonstrated in vitro using both ER (+) MCF-7 and TNBC cell lines. Future studies should explore the effects of this combination across various breast cancer subtypes, including those with specific genetic or molecular profiles, which could provide important insights into its broader applicability and therapeutic potential in personalized medicine. Additionally, investigating the in vivo efficacy of this combination therapy will be crucial for assessing its potential for clinical translation.

## Data Availability

Data and materials are available from the authors upon request. No datasets were generated or analysed during the current study.
